# Prognostic signature of lipid metabolism associated LncRNAs predict prognosis and treatment of lung adenocarcinoma

**DOI:** 10.3389/fonc.2022.986367

**Published:** 2022-11-01

**Authors:** Jie Zhao, Guangjian Li, Guangqiang Zhao, Wei Wang, Zhenghai Shen, Yantao Yang, Yunchao Huang, Lianhua Ye

**Affiliations:** ^1^ Department of Thoracic Surgery, The Third Affiliated Hospital of Kunming Medical University (Yunnan Cancer Hospital), Kunming, China; ^2^ Department of Thoracic Surgery, Taihe Hospital (Hubei University of Medicine), Shiyan, China

**Keywords:** lung adenocarcinoma, lncRNA, prognostic, lipid metabolism genes, signature

## Abstract

**Background:**

Lung adenocarcinoma (LUAD) is the most predominant histological subtype of lung cancer. Abnormal lipid metabolism is closely related to the development of LUAD. LncRNAs are involved in the regulation of various lipid metabolism-related genes in various cancer cells including LUAD. Here, we aimed to identify lipid metabolism-related lncRNAs associated with LUAD prognosis and to propose a new prognostic signature.

**Methods:**

First, differentially expressed lncRNAs (DE-lncRNAs) from the TCGA-LUAD and the GSE31210 dataset were identified. Then the correlation analysis between DE-lncRNAs and lipid metabolism genes was performed to screen lipid metabolism-related lncRNAs. Cox regression analyses were performed in the training set to establish a prognostic model and the model was validated in the testing set and the validation set. Moreover, The role of this model in the underlying molecular mechanisms, immunotherapy, and chemotherapeutic drug sensitivity analysis was predicted by methods such as Gene Set Enrichment Analysis, immune infiltration, tumor mutational burden (TMB), neoantigen, Tumor Immune Dysfunction and Exclusion, chemosensitivity analysis between the high- and low-risk groups. The diagnostic ability of prognostic lncRNAs has also been validated. Finally, we validated the expression levels of selected prognostic lncRNAs by quantitative real-time polymerase chain reaction (qRT-PCR).

**Results:**

The prognostic model was constructed based on four prognostic lncRNAs (LINC00857, EP300-AS1, TBX5-AS1, SNHG3) related to lipid metabolism. The receiver operating characteristic curve (ROC) and Kaplan Meier (KM) curves of the risk model showed their validity. The results of Gene Set Enrichment Analysis suggested that differentially expressed genes in high- and low-risk groups were mainly enriched in immune response and cell cycle. There statistical differences in TMB and neoantigen between high- and low-risk groups. Drug sensitivity analysis suggested that patients with low risk scores may have better chemotherapy outcomes. The results of qRT-PCR were suggesting that compared with the normal group, the expressions of EP300-AS1 and TBX5-AS1 were down-regulated in the tumor group, while the expressions of LINC00857 and SNHG3 were up-regulated. The four prognostic lncRNAs had good diagnostic capabilities, and the overall diagnostic model of the four prognostic lncRNAs was more effective.

**Conclusion:**

A total of 4 prognostic lncRNAs related to lipid metabolism were obtained and an effective risk model was constructed.

## Introduction

Lung cancer is the malignant tumor with the highest morbidity and mortality in the world. There are about 22 million new cases and 17.9 million deaths per year (1, 2). Among them, 85% are non-small cell lung cancer (NSCLC). Lung adenocarcinoma (LUAD) is the most common subtype of NSCLC, accounting for about 40% of all lung cancer subtypes, with characteristics of rapid progression, poor prognosis, and easy recurrence. In recent years, with the continuous application of molecular targeted therapy and immunotherapy, the overall survival rate (OS) has been improved to a certain extent (3). There is an urgent need to find new biomarkers that can effectively predict LUAD.

Long non-coding RNAs (lncRNAs) regulate gene expression through a variety of mechanisms, including transcriptional regulation, translation, protein modification, and activity regulation (4–8). As an important component of cellular biofilms and components, lipids are also involved in energy storage, metabolism, and cell activity signaling molecule transmission. The regulation of cellular processes such as cell growth, differentiation, inflammation, apoptosis, and drug resistance is inseparable from the extensive participation of lipid metabolism (9–11). Therefore, lipid metabolism regulation is crucial for maintaining cellular homeostasis. LncRNAs play a role in lipid metabolism through their effects on SREBP transcription factors, apolipoproteins, triglyceride metabolism, and macrophage cholesterol uptake and efflux (10, 12–15). It has been found that the lncRNA NEAT1 disrupted hepatocellular carcinoma lipolysis by regulating adipose triglyceride lipase, thereby driving hepatocellular carcinoma proliferation (16).In conclusion, LncRNAs are involved in the regulation of various lipid metabolism-related genes in cancer cells (17–21).

Studies have revealed that lncRNA MUC5B-AS1 is up-regulated in lung adenocarcinoma tissues, promotes cell migration and invasion by forming RNA-RNA duplexes with MUC5B (22). The plasma lncRNA H19 level was upregulated in LUAD patients which was correlated with clinicopathological characteristics and had a certain value in lung cancer diagnosis and could assist traditional tumor markers in lung cancer diagnosis and disease evaluation. Wang G et al. performed scRNA-seq detection on early-stage NSCLC and found that there were overall abnormalities in lipid metabolism in different cell types, of which glycerophospholipid metabolism was the most severely altered in lipid metabolism-related pathways (23). The relationship between abnormal lipid metabolism and LUAD has been confirmed by numerous studies. Phosphatidylcholine (PC) and phosphatidylethanolamine (PE) levels were significantly higher in LUAD patients than in healthy individuals (24). High density lipoprotein cholesterol (HDL-C), low density lipoprotein (LDL) and low density lipoprotein receptor (LDLR), sphinolipin, phosphatidylinositol, phosphatidylserine, phosphatidylethanolamine, phospholipid, and phosphatidylcholine are all abnormally expressed in LUAD (25–27).

At present, it is worthwhile to further study whether lipid-metabolism-related lncRNAs may be biomarkers for LUAD. To explore more effective biomarkers in LUAD and explore the potential molecular mechanism of novel lncRNAs in LUAD. In this study, based on The Cancer Genome Atlas (TCGA) database and Gene Expression Omnibus (GEO) datasets, lipid metabolism-related lncRNAs associated with LUAD prognosis were searched. A risk model was established and validated to explore the impact of the risk model on immunotherapy and chemotherapy in patients with LUAD.

## Materials and methods

### Data source

LUAD-related datasets were downloaded from the Cancer Genome Atlas (TCGA) database (https://portal.gdc.cancer.gov/) and the Gene Expression Omnibus (GEO) database (https://www.ncbi.nlm.nih.gov/gds). The TCGA-LUAD dataset contains 59 normal samples and 514 cancer samples. Among these 514 cancer samples, 500 cancer samples have complete survival data and they were divided into a training set (350 samples) and a testing set (150 samples) randomly according to a ratio of 7:3. Moreover, two LUAD-related datasets (GSE31210, GSE50081) were downloaded from the GEO database. Among them, the GSE31210 dataset (containing 20 normal samples and 226 cancer samples) was used for differential analysis. The GSE50081 dataset (containing 181 cancer samples with complete survival data) was used as the validation set. The clinicopathology Characteristics of the TCGA-LUAD cohort, GSE50081 dataset and GSE31210 dataset were shown in **Table 1** Lipid-specific keywords (fatty acyl, glycerolipid, glycerophospholipid, sphingolipid, sterol lipid, prenol) were searched on the Kyoto Encyclopedia of Genes and Genomes (KEGG)website(http://www.kegg.jp/blastkoala/) and the Molecular Signatures Database (MisDB) website (https://www.gsea-msigdb.org/gsea/msigdb/index.jsp) (28).

**Table 1:** The clinicopathology Characteristics of the TCGA-LUAD cohort, GSE50081 dataset, and 110 GSE31210 dataset.

Characteristics	TCGA-LUAD cohort (N=328)	GSE50081 dataset (N=181)	GSE31210 dataset (N=226)
gender			
female	169 (51.52%)	83 (45.9%)	50 (44.2%)
male	159 (48.48%)	98 (54.1%)	105 (46.5%)
age (years)			
>=60	235 (71.65%)	154 (85.1%)	130 (57.5%)
<60	93 (28.35%)	27 (14.9%)	96 (42.5%)
M			
M0	307 (93.60%)	181 (100%)	–
M1	21 (6.40%)	0 (0.0%)	–
N-			
N0	205 (62.50%)	129 (71.3%)	–
N1	71 (21.65%)	52 (28.7%)	–
N2	51 (15.55%)	0 (0.0%)	–
N3	1 (0.30%)	0 (0.0%)	–
T			
T1	100 (30.49%)	57 (31.5%)	–
T2	184 (56.10%)	122 (67.4%)	–
T3	27 (8.23%)	2 (1.1%)	–
T4	17 (5.18%)	0 (0.0%)	–
STAGE			
Stage I	166 (50.61%)	127 (70.2%)	69 (61.1%)
Stage II	81 (24.70%)	54 (29.8%)	58 (25.7%)
Stage III	60 (18.29%)	0 (0.0%)	0 (0.0%)
Stage IV	21 (6.40%)	0 (0.0%)	0 (0.0%)

### Differential expression analysis and correlation analysis

Differential expression analysis was performed with the limma package (29). The DE-lncRNAs between LUAD samples and normal samples in the TCGA-LUAD dataset and the DE-lncRNAs between LUAD samples and normal samples in the GSE312110 dataset were analyzed. FDR< 0.05 and | ${\text{log}}_{2}^{\text{FC}}$ | > 0.5 were considered as a significant difference. The DE-lncRNAs of the two datasets were intersected to screen for common DE-lncRNAs. The correlation between lipid metabolism-related genes and common DE-lncRNAs in the TCGA dataset was calculated. Similarly, the correlation of lipid metabolism-related genes and common DE-lncRNAs in the GSE31210 dataset was also analyzed. |cor| > 0.4 and *P*< 0.05 was considered to have relevance.

### Construction and validation of the prognostic signature

The independent prognostic Lipid metabolism-related DE-lncRNAs were screened by Cox regression analysis (univariate and multivariate) to construct the prognostic features (30). Based on the median risk score (calculated by the expression level of prognostic genes), All patients were divided into two groups(high- and low-risk). The Kaplan-Meier (KM) survival curve was plotted, and the area under curve (AUC) of the receiver operating characteristic (ROC) curve was applied to verify the predictive accuracy. Moreover, the clinical value of the signature was analyzed and the assessment and validation of the risk model were performed in the testing set and validation set.

### Development and assessment of a nomogram

Cox regression analyses(univariate and multivariate) were implemented on the signature and clinical data involving age, gender, and stage (29). We constructed a prediction nomogram based on all independent predictors. And the predictivity of the nomogram was validated using ROC and calibration curves.

### Analysis of potential regulatory mechanisms of prognostic lncRNAs

We calculated the correlation between prognostic lncRNA and mRNA in the TCGA dataset to explore the relevant molecular mechanisms (29). mRNAs with |cor| > 0.7 and *P*< 0.05 were considered as prognostic lncRNA-related mRNAs. Moreover, the Gene Ontology (GO) and Kyoto Encyclopedia of Genes and Genomes (KEGG) enrichment analyses were done to reveal the potential functions of the prognostic genes. Furthermore, a protein-protein interaction network (PPI) (Confidence=0.4) was constructed using the Search Tool for Recurring instances of Neighbouring Genes (STRING) website (https://string-db.org). The survival analysis of the hub gene was carried out. Finally, the DEGs between the two groups were further functionally annotated using Gene Set Enrichment Analysis (GSEA) by Cluster Profiles package in R language.

### Immune infiltration analysis and differences in response to immunotherapy

The linear support vector regression method in CIBERSORT was used to deconvolve the tumor tissue expression matrix to analyze the content of various types of immune cells in the tissue. The rank-sum test was used to analyze the differences in various immune cell contents between the two groups, and the Tumor Immune Dysfunction and Exclusion (TIDE) score, PD-L1, PD-1 distribution, T cell dysfunction score, and T cell exclusion score distribution of each sample (31).

### Differences in TMB levels, neoantigen levels, and chemotherapy drug sensitivity

TMB-the total number of somatic mutations per DNA megabase (Mb) of tumor tissue. TMB per MB was calculated by dividing the total number of mutations by the size of the target coding region. The TMB values in the high- and low-risk groups were calculated separately and then the rank-sum test was carried out. The neoantigen indicators of the samples in the high- and low-groups were extracted from the TCGA database (https://gdc.cancer.gov/about-data/publications/panimmune) and performed tank-sum test. We used the pRRopheticPredict package to analyze the sensitivity of Commonly used drugs for the treatment of LUAD in the Genomics of Drug Sensitivity in Cancer (GDSC) database. Drug sensitivity is represented by the IC50 value.

### Clinical tissue collection

We recruited ten LUAD patients at the Third Affiliated Hospital of Kunming Medical University and collected lung cancer tissue and paracancerous tissue samples from the patients. All participants were exempted from signing informed consent, and this study was reviewed by the Third Affiliated Hospital of Kunming Medical University ethics committee.

### Diagnostic value analysis and expression validation of prognostic lncRNAs

Expression level validation of prognostic lncRNAs was performed in TCGA and GSE31210 datasets, respectively. Para-cancerous tissue and cancerous tissue samples from 10 different LUAD patients were collected, and qRT-PCR was used to verify the expression levels of prognostic genes (32). All tissue was lysed with TRIzol^®^ reagent (Ambion by life technologies, USA, cat:356281), and total RNA was extracted following the manufacturer’s instructions. The extracted RNA was reverse-transcribed to cDNA using the Script RT I First strand cDNA SynthesisAll-in-OneTM First-Strand cDNA Synthesis Kit (cat: G33330-50) before qRT-PCR. The qRT-PCR reaction consisted of 3 µl of reverse transcription product, 5 µl of 2xUniversal Blue SYBR Green qPCR Master Mix (cat: G3326-05), and 1 µl each of forward and reverse primer. PCR was performed in a BIO-RAD CFX96 Touch TM PCR detection system (Bio-Rad Laboratories, Inc., USA) under the following conditions: initial denaturation at 95°C for 1 min, followed by 40 cycles that each involved incubation at 95°C for 20 s, 55°C for 20 s, and 72°C for 30 s. The detailed forward and reverse primer is shown in **Supplementary Table 9**. All primers were synthesized by Servicebio (Servicebio, Wuhan, China). The GAPDH gene served as an internal control, and the relative expression of four lncRNAs was determined using the 2^-ΔΔCt^ method. The experiment was repeated in triplicate on independent occasions. Statistical differences in the four lncRNAs between Para-cancerous tissue and cancerous tissues samples were detected by paired t-test using GraphPad Prism V6 (GraphPad Software, La Jolla, CA, USA), and the level of statistical significance was tested and expressed * as *P*< 0.05, ** means *P*< 0.01. Then, according to the expression levels of prognostic LncRNAs in each dataset, ROC curves of individual LncRNAs and all LncRNAs were drawn.

### Statistical analysis

The statistical analyses in this study were all generated by R software. Wilcoxon test was used to perform a difference comparison between the two groups. Associations between risk scores and gene function or related pathways were calculated by Pearson correlation. Cox regression analysis was used to examine the prognostic power of prognostic features. KM survival analysis and the Cox proportional hazards model were used to analyze the association between the two risk stratifications with the R package Survival. *P*-values less than 0.05 were statistically significant.

## Results

### Differential expression analysis

**Figure 1:**
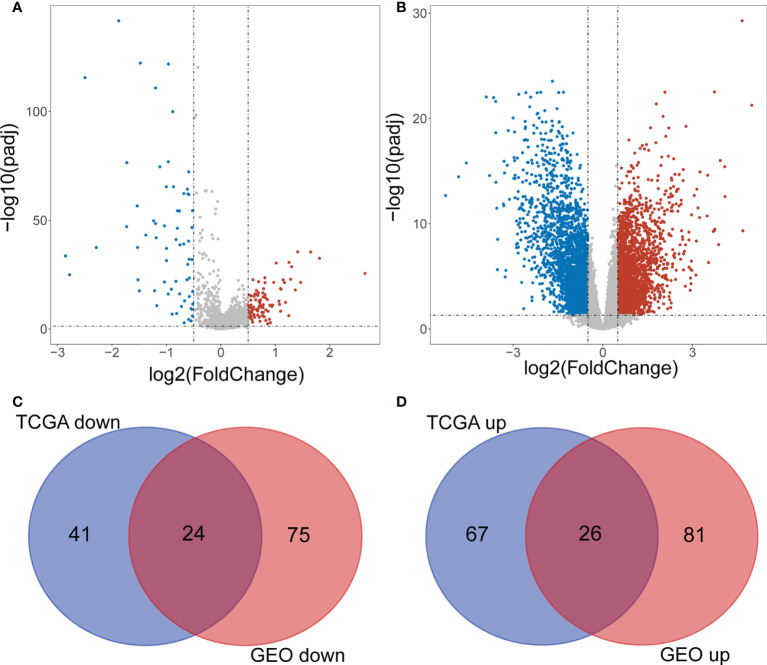
Screening differentially expressed IncRNAs. **(A)** Volcano plot of DE-IncRNAs from TCGA dataset. Red and green indicate up-regulated and down-regulated IncRNAs respectively. **(B)** Volcano plot of DE-IncRNAs from GEO dataset. Red and green indicate up-regulated and down-regulated IncRNAs respectively. **(C, D)** Venn diagram of the intersection of DE-IncRNAs.

The flowchart of the present study was displayed in **Supplementary Figure 1**. A total of 158 DE-lncRNAs(93 were upregulated and 65 were downregulated) were identified from the TCGA dataset(**Figure 1A**). The heat map of the top 100 DE-lncRNAs were shown in **Supplementary Figure 2A**. While a total of 206 DE-lncRNAs(97 were upregulated and 99 were downregulated) were identified from the GEO dataset (**Figure 1B**). The heat map of the top 100 DE-lncRNAs were shown in **Supplementary Figure 2B**. Finally, 50 overlapping lncRNAs were extracted (**Figures 1C, D**).

### Identification of common differentially expressed lipid metabolism lncRNAs

**Figure 2:**
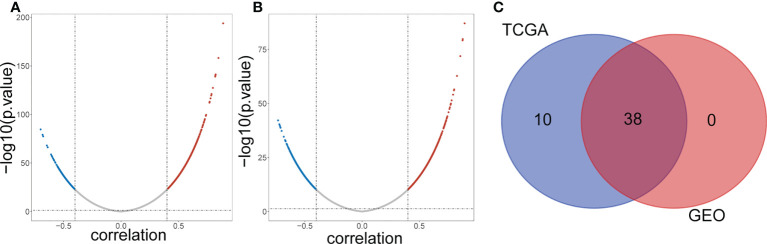
Screening lipid metabolism-related differential expressed IncRNAs. **(A)** Volcano plot of lipid metabolism-related DE-IncRNAs from TCGA dataset. Red and green indicate up-regulated and down-regulated IncRNAs respectively. **(B)** Volcano plot of lipid metabolism-related DE-IncRNAs from GEO dataset. Red and green indicate up-regulated and down-regulated IncRNAs respectively. **(C)** Venn diagram of the intersection of lipid metabolism-related DE-IncRNAs.

A total of 1045 genes related to lipid metabolism were downloaded according to the literature (**Supplementary Table 1**). The expression levels of 50 common DE-lncRNAs and 1011 lipid metabolism-related genes in TCGA samples were extracted, the results of the correlation analysis are shown in **Figure 2A** and **Supplementary Table 2**. A total of 48 lipid metabolism-related DE-lncRNAs were obtained in the TCGA dataset. The correlation of 971 lipid metabolism-related genes and 50 common DE-lncRNAs in the GSE31210 dataset was also analyzed (**Figure 2B** and **Supplementary Table 3**). A total of 38 lipid metabolism-related DE-lncRNAs were obtained in the GSE31210 dataset. After taking the intersection, we obtained 38 common lipid metabolism-related DE-lncRNAs (**Figure 2C**).

### Construction of risk feature

**Figure 3:**
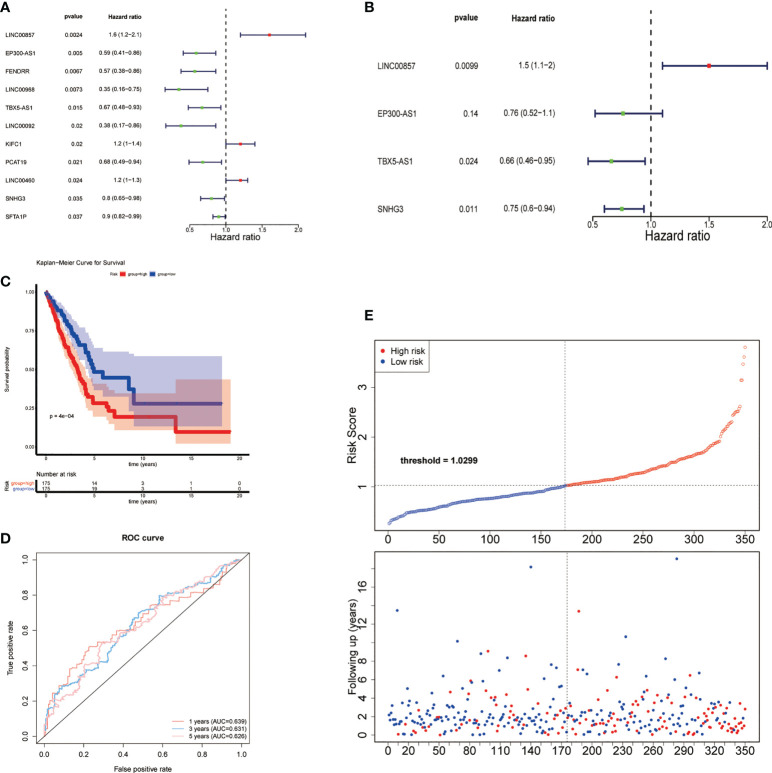
Construction of risk feature. **(A)** Forest plot of univariate Cox regression analysis results of lipid metabolism-related DE-lncRNAs. Red and green indicate risk and protective factors, respectively. **(B)** Forest plot of multivariate Cox regression analysis results of lipid metabolism-related DE-lncRNAs. **(C)** K–M survival curve of the risk score. **(D)** Time-ROC curve analysis of risk feature in 1, 3,5 years. **(E)** The distribution of risk scores, patient survival status, and survival time.

Univariate Cox regression analysis showed that 11 common DE-lncRNAs were significantly related to OS (*P*< 0.05, **Table 2** and **Figure 3A**).

**Table 2:** 11 common DE-lncRNAS were identified by univariate Cox regression analysis.

id	z	HR	HR.95L	HR.95H	pvalue
LINC00857	3.03064	1.56222	1.170701	2.084675	0.00244
EP300-AS1	-2.80409	0.594574	0.413412	0.855123	0.005046
FENDRR	-2.71124	0.574403	0.384727	0.85759	0.006703
LINC00968	-2.68167	0.347052	0.160133	0.752157	0.007326
TBX5-AS1	-2.42441	0.667035	0.480824	0.925363	0.015333
LINC00092	-2.333	0.384128	0.171946	0.858142	0.019648
KIFC1	2.320891	1.200048	1.028765	1.399848	0.020293
PCAT19	-2.30929	0.677321	0.486614	0.942767	0.020927
LINC00460	2.250591	1.15256	1.018504	1.30426	0.024411
SNHG3	-2.10824	0.797356	0.645984	0.984199	0.03501
SFTA1P	-2.08702	0.904885	0.823814	0.993934	0.036887

Subsequently, a new prognostic signature involving 4 lncRNAs (LINC00857, EP300-AS1, TBX5-AS1, SNHG3) was established after multivariate Cox regression analysis (**Table 3** and **Figure 3B**). We calculated risk scores with the following equation: risk score = LINC00857*1.469417445 + EP300-AS1*0.757747569 + TBX5-AS1*0.660575856 + SNHG3*0.752442869. Then separated all samples into two groups depending on the median risk score, (n = 175 and 175, respectively). The patients with high-risk scores had a significantly shorter OS (**Figure 3C**). The AUCs were 0.639, 0.631, and 0.626 at 1, 3, and 5 years, respectively (**Figure 3D**), The distribution of risk scores, patient survival status, and survival time are shown in **Figure 3E**.

**Table 3:** 4 prognostic signatures were identified by multivariate Cox regression analysis.

id	coef	HR	HR.95L	HR.95H	pvalue
LINC00857	0.384866	1.469417	1.096862	1.968513	0.00989
EP300-AS1	-0.2774	0.757748	0.523888	1.096001	0.140707
TBX5-AS1	-0.41464	0.660576	0.460979	0.946595	0.023885
SNHG3	-0.28443	0.752443	0.604604	0.936431	0.010821

### Testing and validation

**Figure 4:**
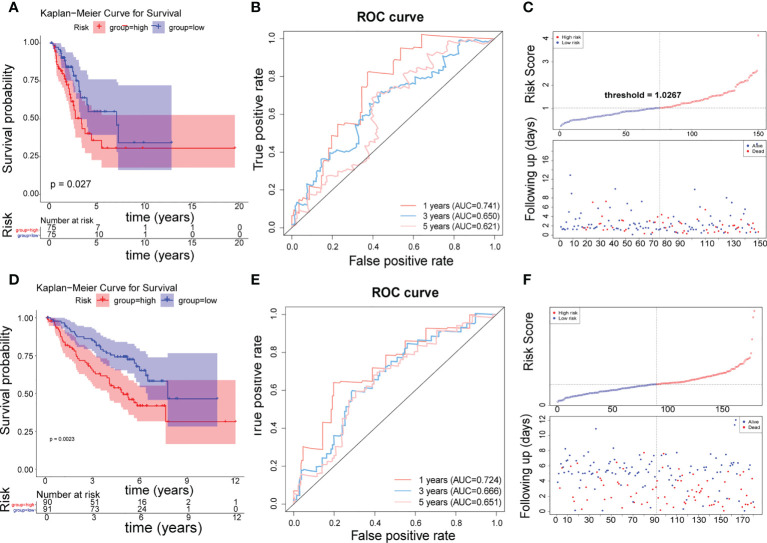
Testing and validation of risk features. **(A)** K–M survival curve of the risk score in the testing set. **(B)** Time-ROC curve analysis of risk feature in 1, 3,5 years in the testing set. **(C)** The distribution of risk scores, patient survival status, and survival time in the testing set. **(D)** K–M survival curve of the risk score in the validation set. **(E)** Time-ROC curve analysis of risk feature in 1, 3,5 years in the validation set. **(F)** The distribution of risk scores, patient survival status, and survival time in the validation set.

The 150 LUAD samples in the testing set were separated into high- (n=75) and low-risk (n=75) groups too. The high-risk group has a worse prognosis than the low-risk group (**Figure 4A**). The AUCs for 1-year, 3-year, and 5-year were 0.741, 0.650, and 0.621, respectively (**Figure 4B**). The distribution of risk scores, patient survival status, and survival time is shown in **Figures 4C**. In addition, we also evaluated the correlation between the risk score and clinical traits (**Supplementary Figure 2C**), There is a significant difference in T stage between high- and low-risk groups (*P*=0.00371) (**Table 4**). The validation of the risk feature was done in the GSE50081 dataset. The 181 LUAD samples in the GSE50081dataset were divided into high-(n=90) and low-risk(n=91) groups. The high-risk group has a worse prognosis than the low-risk group (**Figure 4D**). The AUCs for 1-year, 3-year, and 5-year OS were 0.724, 0.666, and 0.651, respectively (**Figure 4E**). The distribution of risk scores, patient survival status, and survival time is shown in **Figures 4F**. There were significant differences in N, stage, and smoking status between high and low risk groups. (*P*=0.0291, *P*=0.0129, *P*=0.0055, **Supplementary Figure 2D** and **Table 5**). Overall, the 4 lncRNAs we detected were prognostic with both the TCGA-LUAD cohort and GSE50081 dataset.

**Table 4:** Characteristics of patients in low and high risk groups in TCGA-LUAD cohort.

Variable	Expression			
	Total (N=102)	high (N=56)	low (N=46)	P-value
gender				
female	53 (52.0%)	26 (46.4%)	27 (58.7%)	0.301
male	49 (48.0%)	30 (53.6%)	19 (41.3%)	
age (years)				
>=60	77 (75.5%)	40 (71.4%)	37 (80.4%)	0.412
<60	25 (24.5%)	16 (28.6%)	9 (19.6%)	
M				
M0	96 (94.1%)	53 (94.6%)	43 (93.5%)	1
M1	6 (5.9%)	3 (5.4%)	3 (6.5%)	
N				
N0	58 (56.9%)	27 (48.2%)	31 (67.4%)	0.15
N1	32 (31.4%)	21 (37.5%)	11 (23.9%)	
N2	12 (11.8%)	8 (14.3%)	4 (8.7%)	
T				
T1	35 (34.3%)	11 (19.6%)	24 (52.2%)	0.00371
T2	59 (57.8%)	41 (73.2%)	18 (39.1%)	
T3	5 (4.9%)	3 (5.4%)	2 (4.3%)	
T4	3 (2.9%)	1 (1.8%)	2 (4.3%)	
STAGE				
Stage I	52 (51.0%)	24 (42.9%)	28 (60.9%)	0.279
Stage II	30 (29.4%)	20 (35.7%)	10 (21.7%)	
Stage III	14 (13.7%)	9 (16.1%)	5 (10.9%)	
Stage IV	6 (5.9%)	3 (5.4%)	3 (6.5%)	

**Table 5:** Characteristics of patients in low and high risk groups GSE50081 dataset.

Variable	Expression			
	Total (N=181)	high (N=90)	low (N=91)	P-value
gender				
female	83 (45.9%)	38 (42.2%)	45 (49.5%)	0.408
male	98 (54.1%)	52 (57.8%)	46 (50.5%)	
age (years)				
>=60	154 (85.1%)	76 (84.4%)	78 (85.7%)	0.975
<60	27 (14.9%)	14 (15.6%)	13 (14.3%)	
M				
M0	181 (100%)	90 (100%)	91 (100%)	0.941
N				
N0	129 (71.3%)	57 (63.3%)	72 (79.1%)	0.0291
N1	52 (28.7%)	33 (36.7%)	19 (20.9%)	
T				
T1	57 (31.5%)	22 (24.4%)	35 (38.5%)	0.0556
T2	122 (67.4%)	66 (73.3%)	56 (61.5%)	
T3	2 (1.1%)	2 (2.2%)	0 (0%)	
STAGE				
STAGE I	127 (70.2%)	55 (61.1%)	72 (79.1%)	0.0129
STAGE II	54 (29.8%)	35 (38.9%)	19 (20.9%)	
smoking				
Current	57 (31.5%)	35 (38.9%)	22 (24.2%)	0.0055
Ex-smoker	79 (43.6%)	37 (41.1%)	42 (46.2%)	
Never	24 (13.3%)	5 (5.6%)	19 (20.9%)	
Unable to determine	21 (11.6%)	13 (14.4%)	8 (8.8%)	

### Independent prognosis analysis

**Figure 5:**
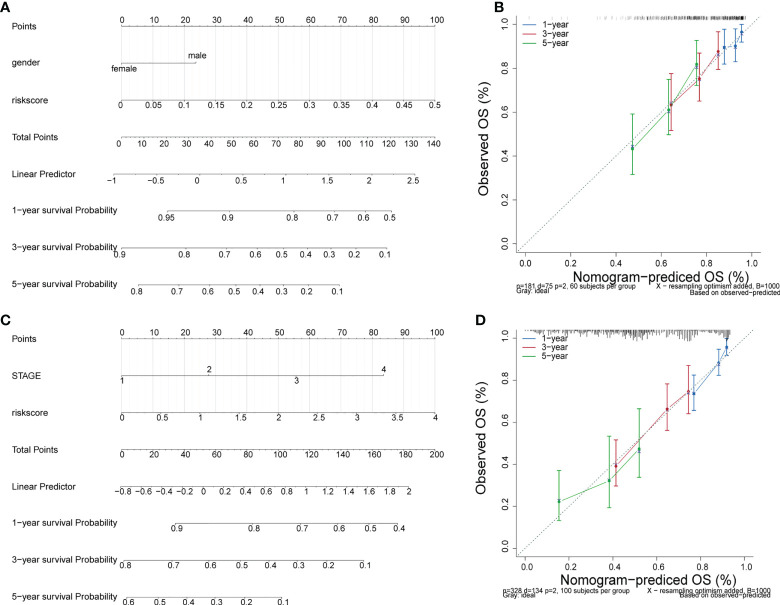
Independent prognosis analysis. **(A)** The nomogram of survival probability based on all independently predictive variables from GEO dataset. **(C)** The nomogram of survival probability based on all independently predictive variables from TCGA dataset. **(B, D)** Calibration curves for evaluating the agreement between the predicted and the actual survival rate for the prognosis model.

Incorporating clinicopathologic data from the TCGA-LUAD cohort into univariate Cox regression analysis, and detected that risk score, gender, M, N, T, and stage were significantly associated with LUAD prognosis (**Table 6**). Subsequently, enrolled these clinicopathological characteristics into multivariate Cox regression analysis and the results indicated that stage and risk score were independent prognostic indicators of LUAD (**Table 7**). Additionally, a nomogram was constructed to predict 1, 3, and 5 years OS of LUAD patients (**Figure 5A**), and the performance of the nomogram was evaluated by calibration curve and demonstrated that 1-, 3-, and 5-year predicted by this nomogram were close to the actual survival duration (**Figure 5B**). Moreover, univariate and multivariate Cox regression analysis were performed and noted that risk score and gender were independent prognostic factors for LUAD patients in the GSE50081 dataset (**Tables 8**, **9**). A prediction nomogram was established and evaluated by calibration curve (**Figures 5C, D**), and showed good predictive accuracy. In summary, all the results indicated that the nomogram based on risk score exhibited a good predictive accuracy for the OS of LUAD patients.

**Table 6:** Prognostic factors associated with LUAD were detected by univariate Cox regression analysis in the TCGA-LUAD cohort.

variable	coef	HR	HR.95L	HR.95H	pvalue
age	0.018574	1.018748	0.994246	1.043854	0.134812
gender	0.659332	1.933501	1.189575	3.142656	0.007804
T	0.743096	2.102435	1.22163	3.618308	0.007304
N	0.428074	1.534299	0.945599	2.489504	0.083015
STAGE	0.523916	1.688628	1.049138	2.717911	0.030967
riskscore	5.500862	244.9029	8.368246	7167.267	0.001407

**Table 7:** Independent prognostic indicators of LUAD were detected by multivariate Cox regression analysis in the TCGA-LUAD cohort.

variable	coef	HR	HR.95L	HR.95H	pvalue
gender	0.650719	1.916919	1.175847	3.12505	0.009065
T	0.516487	1.676129	0.974879	2.881801	0.061769
STAGE	0.418222	1.519259	0.934037	2.471152	0.091984
riskscore	4.243822	69.67362	1.631621	2975.209	0.026722

**Table 8:** Prognostic factors associated with LUAD were detected by univariate Cox regression analysis in the GSE50018 dataset.

variable	coef	HR	HR.95L	HR.95H	pvalue
STAGE	0.459406	1.583133	1.35183	1.854013	1.19E-08
N	0.535674	1.7086	1.405516	2.07704	7.59E-08
T	0.458022	1.580944	1.297362	1.926512	5.60E-06
riskscore	0.504493	1.656146	1.276325	2.148998	0.000147
M	0.608396	1.837482	1.034448	3.263905	0.03794
gender	0.053664	1.05513	0.751376	1.481682	0.756715
age	0.001727	1.001728	0.984147	1.019624	0.84841

**Table 9:** Prognostic factors associated with LUAD were detected by multivariate Cox regression analysis in the GSE50018 dataset.

Variable	coef	HR	HR.95L	HR.95H	pvalue
N	0.22638	1.254052	0.977701	1.608513	0.074683
STAGE	0.250762	1.285005	1.023977	1.612572	0.030427
T	0.213124	1.237538	0.994005	1.540738	0.056626
riskscore	0.361873	1.436016	1.094731	1.883698	0.008957

Because of covering several pages, **Tables 9–11** are provided in **Supplementary Material**.

### Identification of mRNAs associated with prognostic lncRNAs

**Figure 6:**
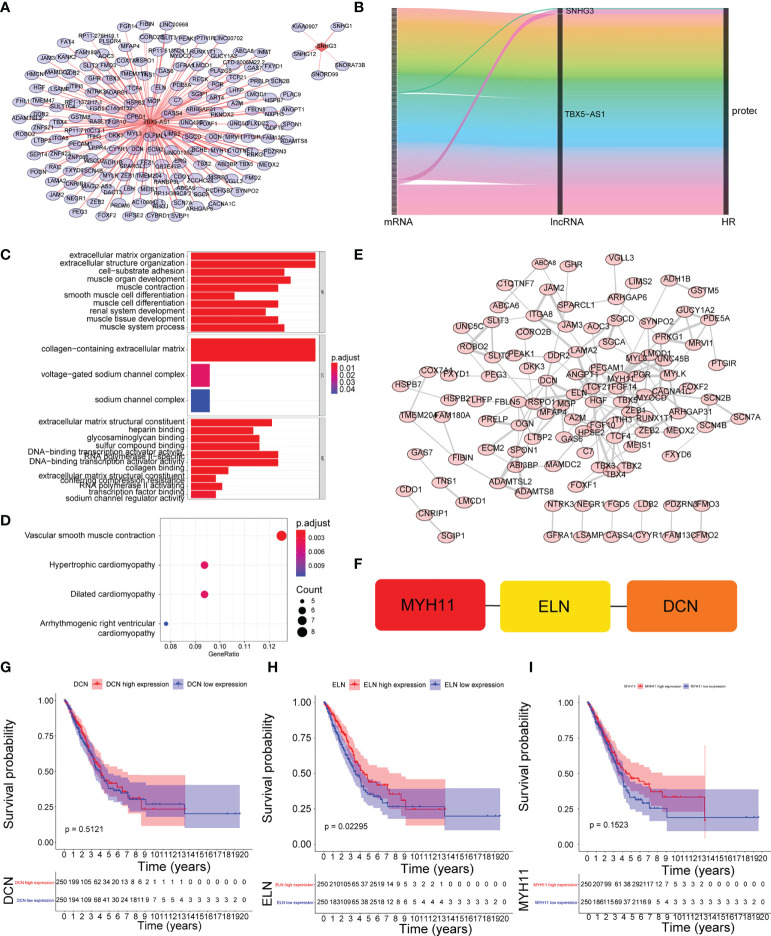
Identification of mRNAs associated with prognostic lncRNAs. **(A)** The coexpression network between prognostic lncRNAs and mRNA. Red diamond nodes represent prognostic lncRNAs, and the sky blue round nodes represent prognostic-related mRNAs. The coexpression network was visualized using Cytoscape 3.7.2 software. Green circles represent mRNAs that are both associated with prognostic lncRNAs and cis-regulated. **(B)** Sankey diagram showed the association between prognostic related lncRNAs and mRNAs.**(C)** GO enrichment analysis. **(D)** KEGG enrichment analysis. **(E)** Protein interaction network. **(F)** identification of the top3 hub genes. **(G–I)** K–M survival curve of the expression levels of MYH11, ELN, DCN and the corresponding survival information.

We also focused on the four prognostic lncRNA-associated mRNAs, but only two of them obtained associated mRNAs, a total of 171 mRNAs associated with the two prognostic lncRNAs were obtained (**Tables 10**, **11**), and their relationships are shown in **Figures 6A**, **B**, with 9 mRNAs were both associated with prognostic lncRNAs and cis-regulated (**Supplementary** **Figure 3**). The GSEA enriched results showed that the prognostic-related lncRNA had a strong correlation with collagen-containing extracellular matrix, extracellular matrix organization, and extracellular matrix structural constituent conferring compression resistance (**Figure 6C**). The key enrichment pathways were “respiratory system development”, “lung development”, and lung alveolus development” (**Figure 6D**). Therefore, these prognostic genes may affect the prognosis of LUAD patients by regulating the structural changes of the extracellular matrix or affecting the development of the respiratory system. The detailed GSEA results are shown in **Supplementary Table 4** and **Table 5**. A 109-protein interaction network was constructed by the STRING (https://string-db.org) website (**Table 12** and **Figure 6E**). After identification of hub genes by cytohubba function of Cytoscape arrayed the hub genes according to MCC (**Supplementary Table 6**). Finally, the top3 hub genes (MYH11, ELN, DCN) were obtained (**Figure 6F**). Moreover, the expression levels of MYH11, ELN, DCN and the corresponding survival information were extracted for survival analysis (**Figures 6G–I**). The results showed that patients with higher ENL gene expression had an optimistic prognosis (*P*=0.02295), while there was no significant difference in survival between the high and low expression groups of MYH11 and DCN.

### GSEA

**Figure 7:**
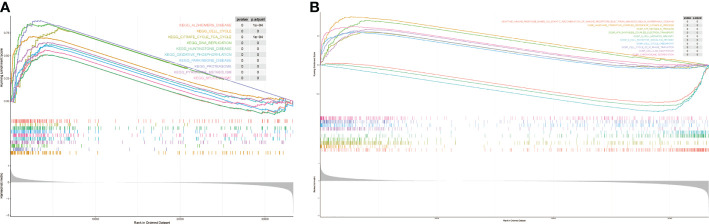
The results of gene set enrichment analysis. **(A)** KEEG enrichment analysis. **(B)** GO enrichment analysis.

We performed GSEA to explore the potential mechanisms of the risk model. the enriched results showed that the prognostic differences between it may be related to the up-regulation of pathways such as DNA replication, oxidative-phosphorylation, and pyrimidine-metabolism (**Figure 7A**). The key enrichment pathways were “b cell-mediated immunity “,”cell cycle checkpoint”, “chromosome segregation” etc (**Figure 7B**). The detailed results are shown in **Supplementary Tables 7** and **8**. Notably, these pathways were significantly enriched in samples with high risk score.

### Immune infiltration analysis and differences in response to immunotherapy

**Figure 8:**
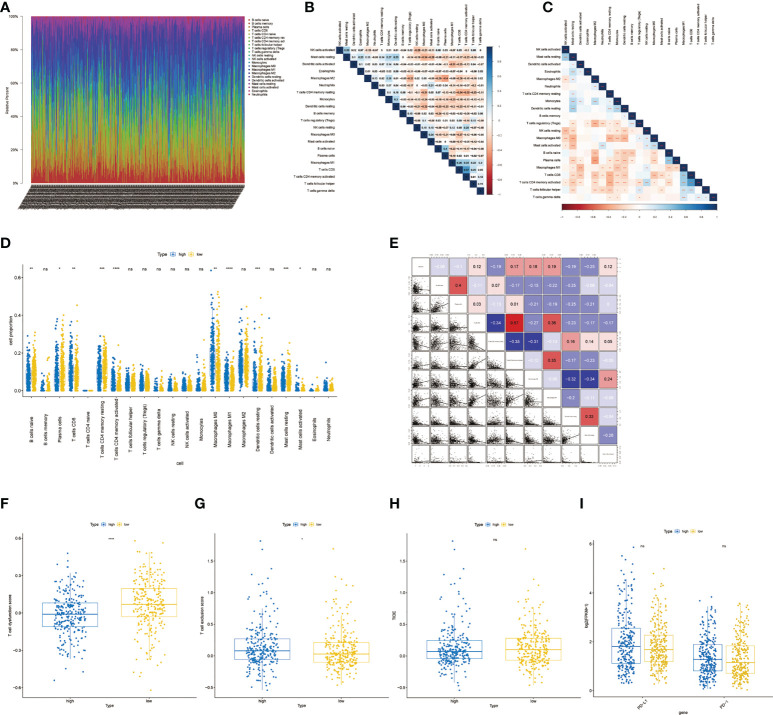
Immune infiltration analysis and differences in response to immunotherapy.**(A)** Proportion of immune cell infiltration.**(B)** Correlation heatmap of immune cell proportion. **(C)** Correlation p-value heatmap of immune cell proportion. **(D)** Boxplot of immune cell differences between the low- and high-risk group. **(E)** The correlation between significantly different immune cells and risk scores. **(F)** Boxplot of T cell dysfunction score between high- and low-risk groups. **(G)** Boxplot of T cell exclusion score between high- and low-risk groups.**(H)** Boxplot of TIDE score between high- and low-risk groups. *, **, ***, and **** represent P < 0.05, P<0.01, P < 0.001, and P < 0.0001, respectively. ns, not significant.

The RNA-seq data of 500 patients with LUAD from the TCGA database were analyzed to evaluate the immune landscape. Marker genes of 22 immune cell species were evaluated between the two groups (**Figures 8A–C**). Then, a rank-sum test was performed, and as shown in the boxplot, 10 of the 22 immune cells showed significant differences (**Figure 8D**). We also calculated the correlation between 10 significantly different immune cells and risk scores (**Figure 8E**). The results showed the expressions of resting memory CD4 T cells, resting dendritic cells, and resting mast cells were negatively correlated with the risk score, while activated memory CD4 T cells, M0 macrophages and M1 macrophages expressions were positively correlated with the risk score. The rank-sum test results showed that there were significant differences in T cell dysfunction score and T cell exclusion score between the two groups (**Figures 8F, G**, *P<* 0.0001 and *P*< 0.05). However, there was no significant differences in TIDE scores, PD-1, and PD-L1 between high- and low-risk groups (**Figures 8H, I**).

### Differences in TMB levels, neoantigen levels and chemotherapy drug sensitivity

**Figure 9:**
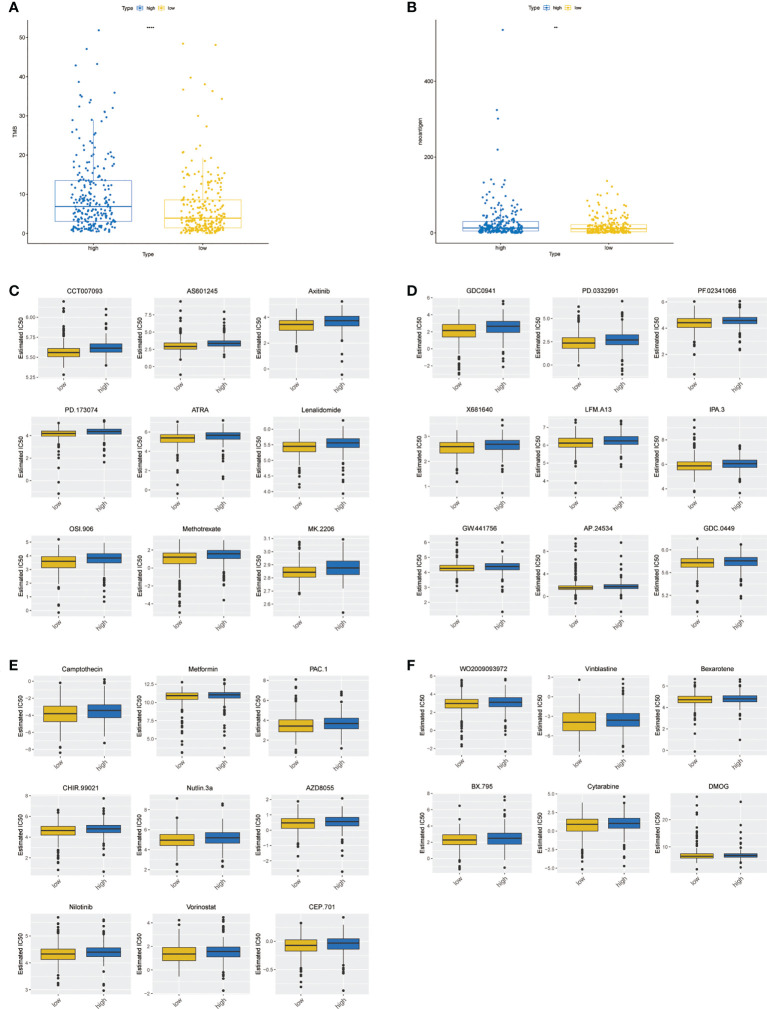
Differences in TMB levels, neoantigen levels, and chemotherapy drug sensitivity between high- and low-risk groups. **(A)** Boxplot of the TMB between high- and low-risk groups. **(B)** Boxplot of the neoantigen values between high- and low-risk groups. **(C–F)** Boxplot of 33 chemotherapy drug sensitivity between high- and low-risk groups. ** represents P < 0.01, **** represents P < 0.0001.

The rank-sum test of the TMB was performed, and the results showed that there was a significant difference between the two groups (**Figure 9A**, *P*< 0.0001). Moreover, the rank-sum test of the neoantigen values was performed, and the results suggested that the neoantigen values of the two groups were significantly different (**Figure 9B**, *P*< 0.01). A total of 33 drugs showed significant differences between the two groups (**Figures 9C–F**). Overall, the IC50 value of the low-risk group was lower, indicating that the chemotherapy effect of patients in the low-risk group may be more optimistic.

### Expression validation and diagnostic value analysis of prognostic lncRNAs

**Figure 10:**
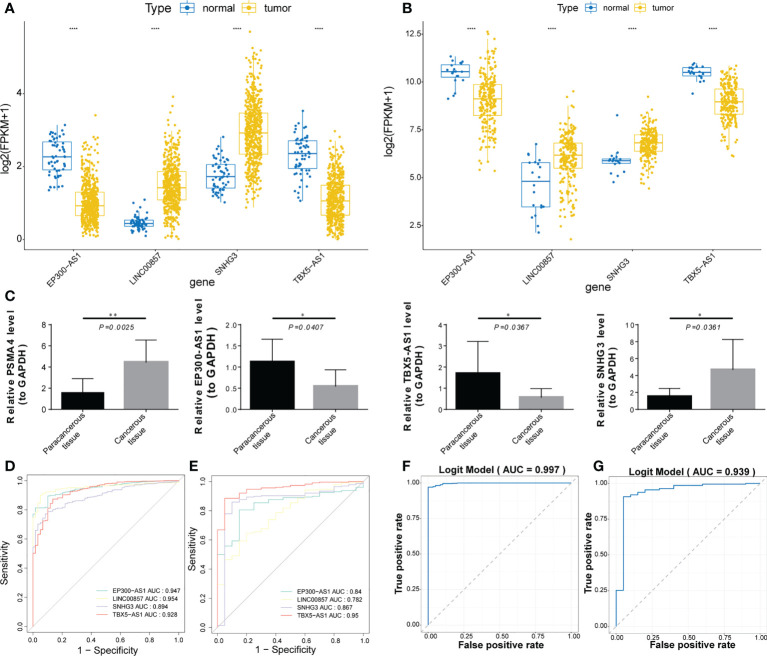
Expression validation and diagnostic value analysis of prognostic lncRNAs. **(A, B)** Boxplot of the expression trends of the four prognostic lncRNAs in 2 datasets. **(C)** Boxplots of the expression levels of the four lncRNAs between the paracancerous and cancerous tissue samples. **(D, E)** ROC curve analysis of prognostic lncRNAs diagnostic ability in 2 datasets. **(F, G)** ROC curve analysis of the overall diagnostic model in 2 datasets. *, **, and **** represent P < 0.05, P < 0.01, and P < 0.0001, respectively.

The expression trends of the four prognostic factors in LUAD and normal samples from the TCGA-LUAD and the GSE31210 datasets were basically consistent. Compared with the normal group, the expressions of EP300-AS1 and TBX5-AS1 was down-regulated in the tumor group, while the expressions of LINC00857 and SNHG3 were up-regulated (**Figures 10A, B**). The qRT-PCR was used to verify the expression levels of prognostic genes and indicated that the expression levels of the four lncRNA were distinctly different between the Para-cancerous tissue and cancerous tissues samples (all P< 0.05; **Figure 10C**), with the expression of LINC00857 (*P* = 0.0025, *t* = 2.388) and SNHG3 (*P* = 0.0361, *t* = 2.462) being up-regulated in the cancerous tissues, the expression of TBX5-AS1 (*P* = 0.0367, *t* = 2.451) and EP300-AS1 (*P* = 0.0407, *t* = 2.388) being down-regulated in the cancerous tissues, consistent with the results of TCGA-LUAD cohort and GSE31210 dataset. Prognostic lncRNAs have a good diagnostic ability in TCGA and GSE31210 datasets, with AUC greater than 0.78 (**Figures 10D, E**). In addition, we also evaluated the overall diagnostic performance of 4 genes, which performed well in TCGA-LUAD cohort (AUC = 0.997) and GSE31210 dataset (AUC = 0.939), with AUC greater than 0.9 (**Figures 10F, G**). This indicates that the overall diagnostic model of the four prognostic lncRNAs is more effective than the single-gene diagnostic effect.

## Discussion

Dysregulation of lipid metabolism is one of the most representative metabolic disorders in cancer. Lipid metabolism is used by cancer cells to enable cancer cells to proliferate, survive, invade, metastasize, and obtain the energy, biofilm components and signal molecules required for cancer microenvironment and cancer treatment response consists of various types of cells, cytokines, growth factors, and nutrients including lipids (33). LncRNAs have become key biomarkers for tumor diagnosis and treatment (34). Numerous studies have focused on the functions of genes involved in lipid metabolism (35–38). There were no studies have been seen on lipid metabolism-related lncRNA features to predict the prognosis and treatment of lung adenocarcinoma patients. Therefore, we conducted this study to establish a lipid metabolism-related lncRNA signature based on a large-scale database to predict the prognosis and treatment of LUAD patients.

In this study, we screened lipid metabolism-related lncRNAs for the first time by correlation analysis between lncRNAs and lipid metabolism-related genes using multiple datasets from the TCGA and GEO cohorts. Prognostic features based on four lipid metabolism-related lncRNAs (LINC00857, EP300-AS1, TBX5-AS1, SNHG3) were constructed using COX regression. And validated by ROC curve and KM curve in an independent cohort, this risk prediction model can be used as an independent prognostic factor for LUAD.

Our study found that four lncRNAs including LINC00857, EP300-AS1, TBX5-AS1, and SNHG3 were abnormally expressed in LUAD. Among them, LINC00857 is overexpressed in LUAD and can regulate the proliferation, apoptosis and glycolysis of LUAD cells by targeting the miR-1179/SPAG5 axis (39). TBX5-AS1 functions in LUAD, lung squamous cell carcinoma (LUSC), Adrenocortical carcinoma, and uterine corpus endometrial carcinoma. TBX5-AS1 belongs to a subclass of lncRNAs called enhancer RNAs. TBX5-AS1 is downregulated in LUAD. Inhibition of tumor progression through the PI3K/AKT pathway affects the prognosis of LUAD patients (40–43). SNHG3 is up-regulated in ovarian cancer, glioma, hepatocellular carcinoma, and osteosarcoma, all of which are associated with poor prognosis (44–47). SNHG3 is overexpressed in lung cancer tissues and cells, and a lot of studies have suggested that SNHG3 can affect the prognosis of LUAD through multiple pathways. For example, SNHG3 was activated by E2F1 and promoted not only proliferation but also the migration of LUAD cells through activating TGF-β pathway and IL-6/JAK2/STAT3 pathway (48). SNHG3 promotes the occurrence and progression of LUAD through regulating miR- 515-5p/SUMO2 axis, miR-216a/ZEB1 axis, the miR-1343-3p/NFIX pathway or the expression of miR-890 (49–52).

In this study, the expression of EP300-AS1 was down-regulated in LUAD, which may play a tumor suppressor role in LUAD. At present, there is no relevant research report, and the specific molecular mechanism and prognostic potential need to be further explored.

Since our model was confirmed to have good predictive accuracy, we performed GSEA enrichment analysis of prognostic lncRNA-related mRNAs, and the results suggested that they could influence the prognosis of LUAD patients by modulating structural changes in the extracellular matrix or affecting the development of the respiratory system. The GO and KEGG analysis showed that it was enriched in multiple biological processes, mainly involved in DNA replication, oxidative phosphorylation, and pyrimidine metabolism. The key enrichment pathways for prognostic features are B-cell mediated immunity, cell cycle checkpoint, chromosomal segregation, etc. We found that this feature is more involved in the biological processes and pathways of the cell cycle and immune response. Studies have shown that lnc00857 can regulate the cell cycle by regulating CCNE1 and CDK2 expression causing G1/S phase arrest (53). Down-regulation of TBX5-AS1 expression improves cell viability, migration, and invasion, while inhibiting apoptosis (40). SNHG3 regulates LUAD cell proliferation and cell cycle while inhibiting apoptosis (54). Based on the above results, the difference in prognosis between high and low risk groups may be related to cell cycle and immune pathways.

Based on enrichment analysis, prognostic features were associated with immune pathways. Finally, the immune infiltration analysis revealed that the expressions of T cells CD4 memory rest, Dendritic cells resting and Mast cells resting were negatively correlated with risk score, while T cells CD4 memory activated, Macrophages M0 and Macrophages M1 expressions were positively correlated with risk score. The predicted prognosis of LUAD patients may be related to differences in immune cell composition.

We used three immunotherapy biomarkers to assess the predictive power of this signature. Notably, we predict the possibility of response to immunotherapy based on TIDE score and used the TIDE to predict immune checkpoint blockade response. It integrates the expression of two main mechanisms of tumor immune escape, T cell dysfunction and T cell rejection, to simulate tumor immune escape and can be used to predict the immunotherapy response of lung cancer (55). Interestingly, the low-risk group had higher T cell dysfunction score, while the high risk group had higher T cell exclusion score. This suggests that high-risk groups may benefit from the administration of checkpoint inhibitors (ICIs). Next, we also compared tumor mutational burden(TMB)and neoantigen between high and low-risk groups. Both TMB and neoantigens play an important role in tumor immune response. TMB can be used as a biomarker of response to checkpoint inhibitors (56), and neoantigens can be used as a biomarker to predict immune response to lung cancer (57). As predicted, the high-risk group exhibited higher mutational loads and neoantigens. Relatively speaking, the high-risk group had a higher objective response rate to immunotherapy and could benefit from the ICIs of LUAD (58). Studies have shown that the ability of TIDE to predict response to immunotherapy has been proven to be empirically superior to known immunotherapy biomarkers such as TMB and neoantigens (55). However, there was no significant difference in TIDE score, PD-1, and PD-L1 distribution between the two groups. Whether this feature can effectively predict the response to immunotherapy needs further research to verify.

We also focused on the differences in chemotherapeutic drug sensitivity. Interestingly, IC50 values in the low-risk group were significantly lower than those in the high-risk group, suggesting that the patients in the low-risk group may have better outcomes with chemotherapy. Based on this result, individualized treatment regimens can be developed according to the risk scores of different LUAD patients.

A large number of studies have shown that lncRNAs have been confirmed to be associated with poor prognosis of cancer, and the expression levels of lncRNAs can be used as diagnostic markers in addition to prognostic markers of intrinsic characteristics of cancer (59). A diagnostic biomarker detects or confirms the presence of a disease or condition of interest, or identifies an individual with a subtype of the disease. However, the diagnostic value of lncRNAs is rarely reported. The development of new diagnostic biomarkers is particularly important for early detection, early treatment and improved prognosis of LUAD patients (60). Therefore, to verify whether our model has diagnostic value, we performed expression validation of prognostic lncRNAs using an external independent dataset, and validated the expression levels of prognostic-related genes by performing qRT-PCR on paracancerous tissue and cancerous tissue samples from LUAD patients. Biomarkers need to ensure a low false positive rate. The use of ROC curve is conducive to the rational use of diagnostic biomarker evaluation. Decision thresholds and clinical utility are becoming important measures for assessing the value of biomarkers for clinical application (61). In this study, four lncRNAs (LINC00857, EP300-AS1, TBX5-AS1, and SNHG3) that are significantly related to the survival of LUAD patients (p<0.05) were used as diagnostic biomarkers, and their false positive concerns as LUAD biomarkers were ruled out by drawing the ROC curve of prediction model and single lncRNA. The results of clinical and independent prognostic analysis show that the risk score based on this model has independent prognostic value, and the corresponding nomogram and calibration curve also show that the model we built can be used in clinical diagnosis. On the other hand, the method of cross validation using multiple mutually exclusive “training” and “validation” samples is usually used for clinical validation of biomarkers (62). In this study, TCGA-LUAD (test set and validation set) and GSE50081 (external validation set) were jointly used to evaluate risk models, ensuring their effectiveness. As expected, the four prognostic lncRNAs signatures related to lipid metabolism showed good diagnostic ability and were able to distinguish between paracancerous and cancerous tissues. At the same time, the overall diagnostic model of the four prognostic lncRNAs is more robust than the individual lncRNAs. This study also suggests that this lncRNAs signature is a potential diagnostic tool for LUAD patients.

Based on survival analysis, prognostic features can effectively predict the total survival (OS) of early LUAD (63). By combining the prognostic features with classic clinical risk factors, it can also be found that when patients are exposed to the same clinical risk factors, the prognosis of the high-risk group is significantly worse. At the same time, prognostic features can also be used to guide treatment (64). A large number of studies have shown that proteins and mRNAs have been validated as biomarkers of various cancers (38, 65). However, the stability of these biomarkers will be affected by the regulation and modification of proteins and mRNAs at the transcriptional level. LncRNAs are effectors whose function depends on their expression levels. Many lncRNAs have also been shown to be associated with poor cancers prognosis (59, 66, 67). Therefore, the expression level of lncRNAs can be used as a better biomarker to predict or diagnose the prognosis of tumors. In this study, this lncRNAs signature is not only related to lipid metabolism, but also related to specific biological mechanisms. In contrast, this is the advantage of our prognostic model.

Although this is the first time to construct the prognostic characteristics of lipid metabolism-related lncRNA of LUAD, and multi-dimensional verification has been carried out, our study still has certain limitations. First, the data analyzed in this study are all from online databases, and larger samples are needed to further study the clinical application of our findings in LUAD. Second, this paper is a retrospective study, needed to corroborate by corresponding prospective studies. Finally, functional experiments are needed to further elucidate the intrinsic molecular mechanisms of lipid metabolism-related lncRNAs.

In conclusion, this study constructed the prognostic characteristics of 4 lncRNAs related to lipid metabolism for the first time by analyzing bioinformatics methods and based on multiple databases, which proved to have important prognostic and therapeutic value for LUAD patients, as well as good diagnostic ability.

## Data availability statement

The original contributions presented in the study are included in the article/**Supplementary Material**. Further inquiries can be directed to the corresponding authors.

## Ethics statement

The studies involving human participants were reviewed and approved by The Third Affiliated Hospital of Kunming Medical University ethics committee. Written informed consent for participation was not required for this study in accordance with the national legislation and the institutional requirements.

## Author contributions

JZ and GL contributed to conception and design of the study. JZ organized the database, performed the statistical analysis and wrote the first draft of the manuscript. GZ, WW, ZS, and YY wrote sections of the manuscript. LY and YH manuscript revision. All authors contributed to read, and approved the submitted version.

## Funding

This work was supported by Yunnan Fundamental Research Projects (No. 202201AY070001-135); National Natural Science Foundation of China (No. 81960500); Young and Middle-aged Academic and Technical Leaders Reserve Talents Project of Yunnan Province (No. C20048).

## Acknowledgments

We appreciated TCGA and GEO databases for providing the original study data.

## Conflict of interest

The authors declare that the research was conducted in the absence of any commercial or financial relationships that could be construed as a potential conflict of interest.

## Publisher’s note

All claims expressed in this article are solely those of the authors and do not necessarily represent those of their affiliated organizations, or those of the publisher, the editors and the reviewers. Any product that may be evaluated in this article, or claim that may be made by its manufacturer, is not guaranteed or endorsed by the publisher.
