# Supplementary material for: Prognostic signature of lipid metabolism associated LncRNAs predict prognosis and treatment of lung adenocarcinoma
Source: Front Oncol. 2022 Nov 1;12:986367. doi: 10.3389/fonc.2022.986367 (PMC9664164; doi:10.3389/fonc.2022.986367)
Supplement: Supplementary file 1 [file DataSheet_1.zip › supplementary table/supplementary table 9.docx]

| **Supplementary Table9 qRT-PCR primer sequences** | |
| --- | --- |
| **LncRNA** | **Sequence** |
| YQXX-0538-H-LINC00857 F | GAAAAGACACCAAACTCGG |
| YQXX-0538-H-LINC00857 R | CTCATACACTCAACCCAGC |
| YQXX-0538-H-EP300-AS1 F | CAGAGCGGGAGGAGCG |
| YQXX-0538-H-EP300-AS1 R | CGGGGGGCAGAGGTTG |
| YQXX-0538-H-TBX5-AS1 F | GTCACTTTCCCTCTCACCG |
| YQXX-0538-H-TBX5-AS1 R | TTCTCATTTCATTACCCCG |
| YQXX-0538-H-SNHG3 F | TGGAGTTTGGTGGACTGTAGA |
| YQXX-0538-H-SNHG3 R | TAGGAAGATGCCTTTTTTTTG |
| internal control H-GAPDH F | CCCATCACCATCTTCCAGG |
| internal control H-GAPDH R | CATCACGCCACAGTTTCCC |
