# Supplementary figures and images for: Prognostic signature of lipid metabolism associated LncRNAs predict prognosis and treatment of lung adenocarcinoma
Source: Front Oncol. 2022 Nov 1;12:986367. doi: 10.3389/fonc.2022.986367 (PMC9664164; doi:10.3389/fonc.2022.986367)

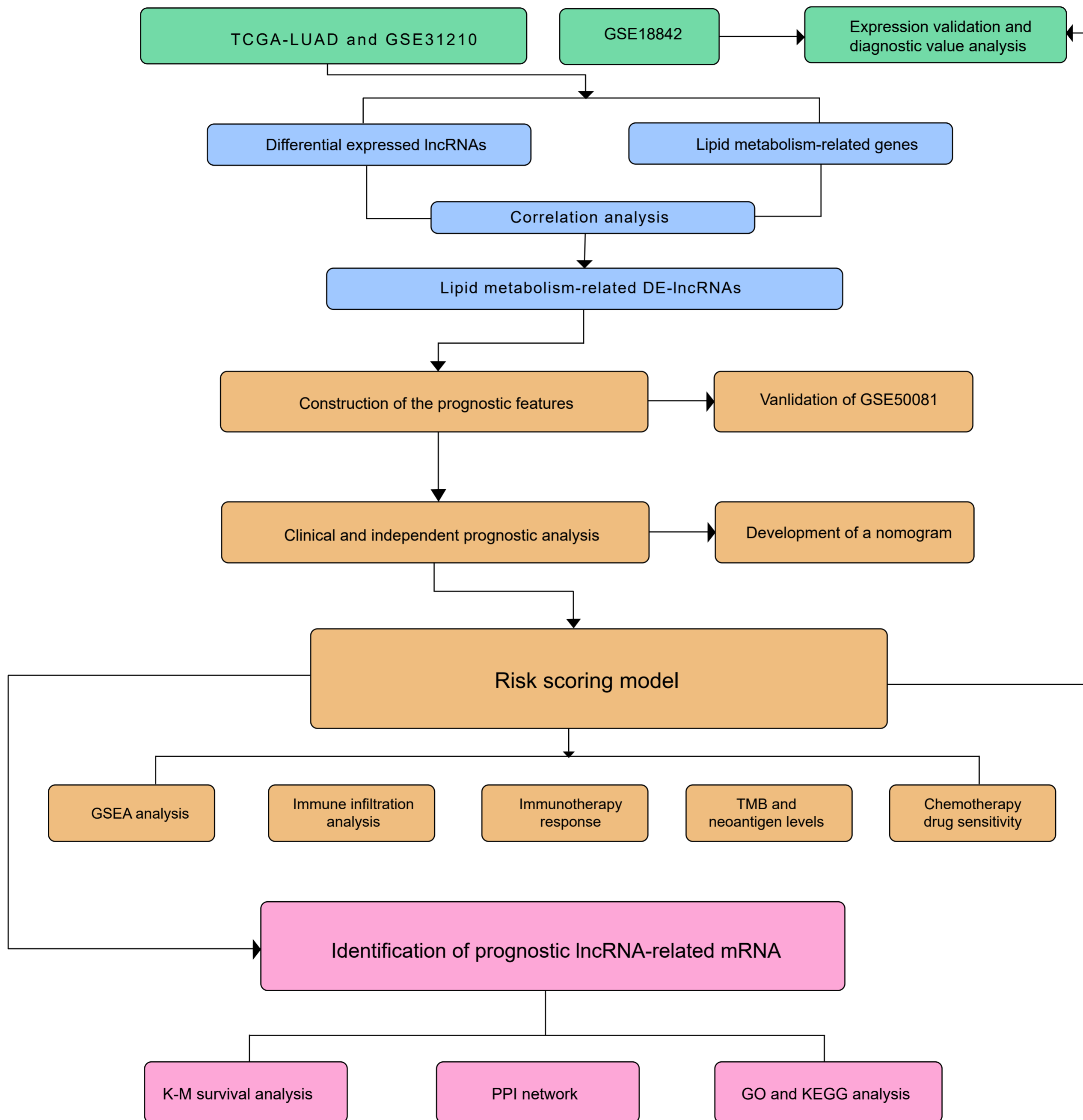

Supplement: Supplementary file 2 [file DataSheet_2.zip › Supplementary Figure/Supplementary Figure 1.pdf]

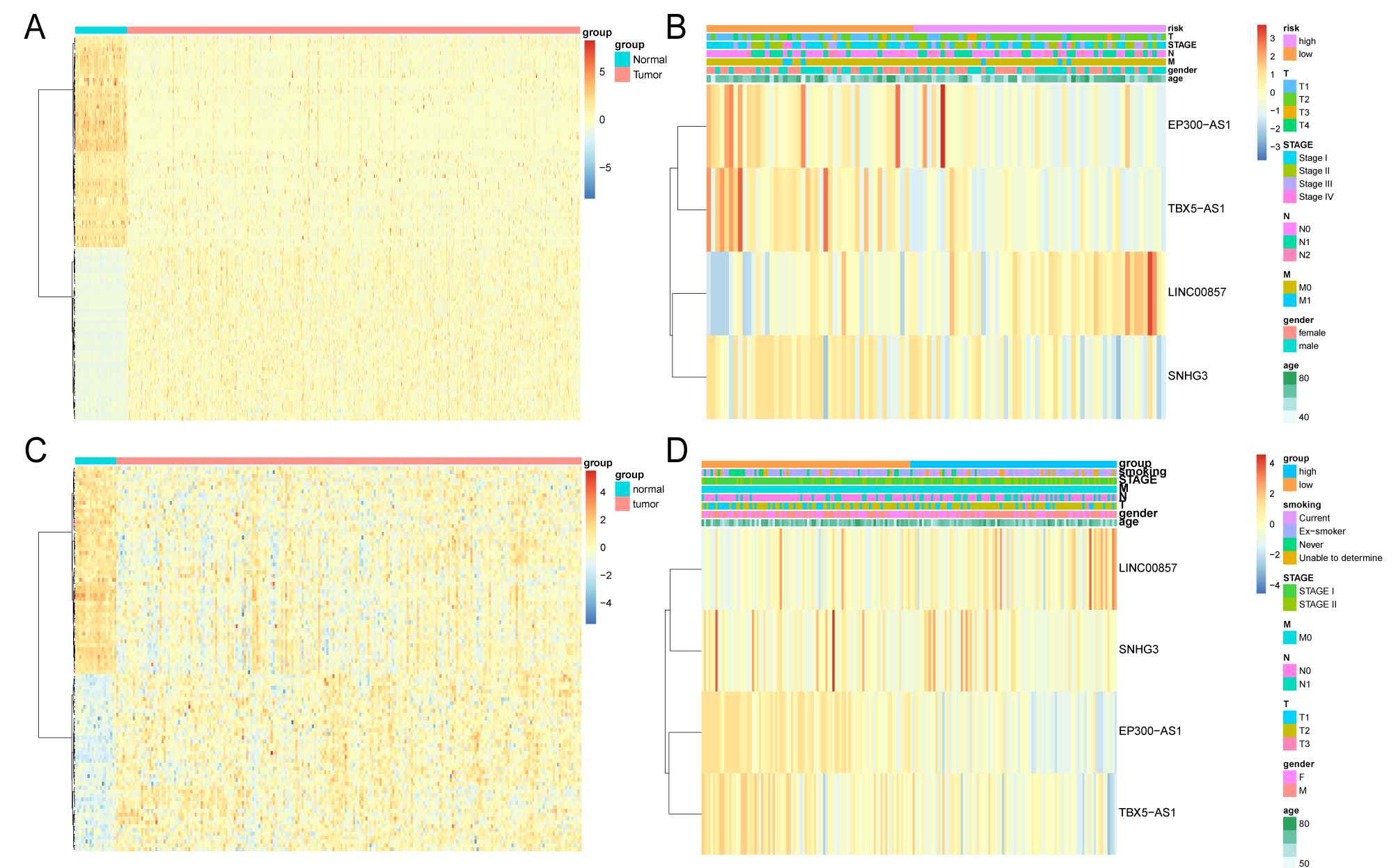

Supplement: Supplementary file 2 [file DataSheet_2.zip › Supplementary Figure/Supplementary Figure 2.tif]

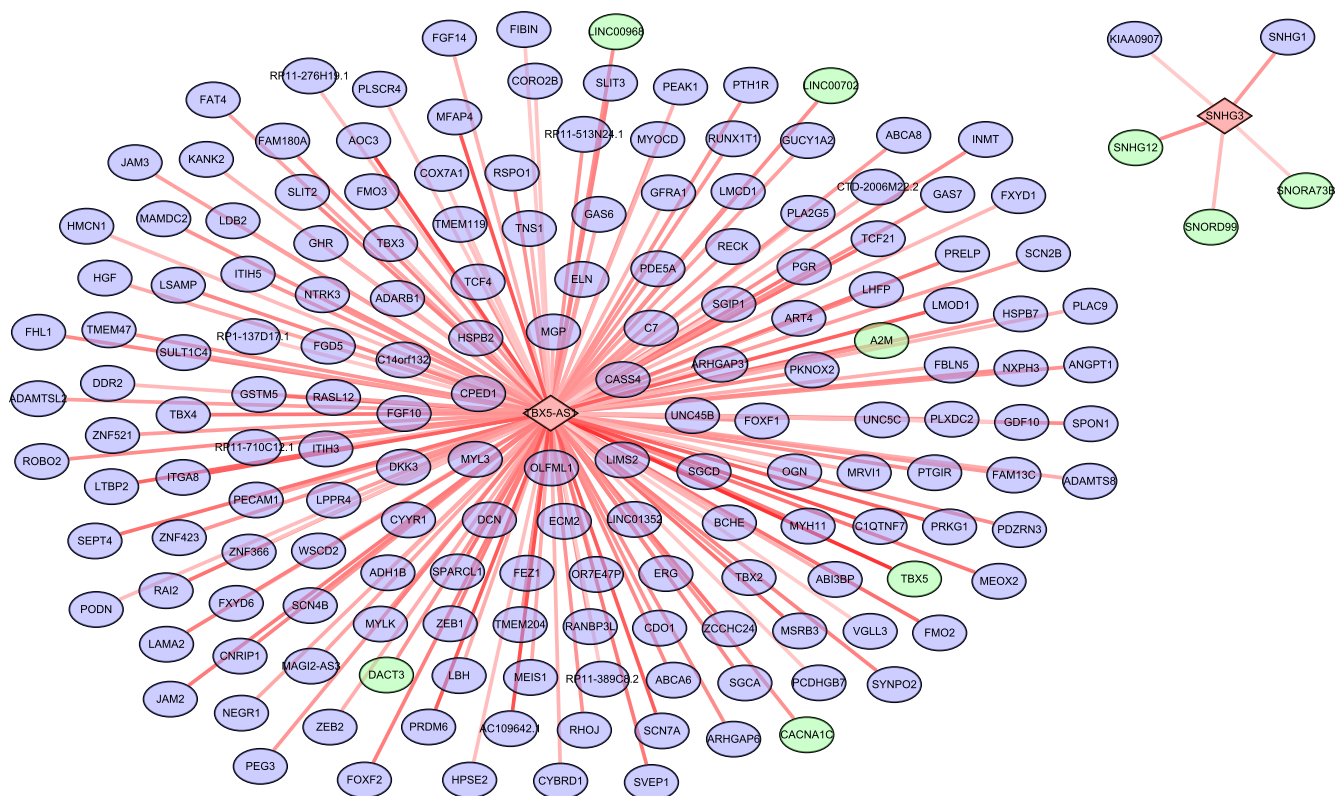

Supplement: Supplementary file 2 [file DataSheet_2.zip › Supplementary Figure/Supplementary Figure 3.pdf]
